# Modulation Spectral Signal Representation for Quality Measurement and Enhancement of Wearable Device Data: A Technical Note

**DOI:** 10.3390/s22124579

**Published:** 2022-06-17

**Authors:** Abhishek Tiwari, Raymundo Cassani, Shruti Kshirsagar, Diana P. Tobon, Yi Zhu, Tiago H. Falk

**Affiliations:** 1 Institut National de la Recherche Scientifique, University of Quebec, Montréal, QC H5A 1K6, Canada; abhishek.tiwari@myant.ca (A.T.); shruti.kshirsagar@inrs.ca (S.K.); yi.zhu@inrs.ca (Y.Z.); 2 Myant Inc., Toronto, ON M9W 1B6, Canada; 3 McConnell Brain Imaging Centre, Montreal Neurological Institute, McGill University, Montréal, QC H3A 2B4, Canada; raymundo.cassani@mcgill.ca; 4 Faculty of Engineering, Universidad de Medellín, Medellín 050026, Colombia; dtobon@udemedellin.edu.co

**Keywords:** modulation spectrum, wearable devices, quality measurement, signal enhancement, feature engineering

## Abstract

Wearable devices are burgeoning, and applications across numerous verticals are emerging, including human performance monitoring, at-home patient monitoring, and health tracking, to name a few. Off-the-shelf wearables have been developed with focus on portability, usability, and low-cost. As such, when deployed in highly ecological settings, wearable data can be corrupted by artifacts and by missing data, thus severely hampering performance. In this technical note, we overview a signal processing representation called the modulation spectrum. The representation quantifies the rate-of-change of different spectral magnitude components and is shown to separate signal from noise, thus allowing for improved quality measurement, quality enhancement, and noise-robust feature extraction, as well as for disease characterization. We provide an overview of numerous applications developed by the authors over the last decade spanning different wearable modalities and list the results obtained from experimental results alongside comparisons with various state-of-the-art benchmark methods. Open-source software is showcased with the hope that new applications can be developed. We conclude with a discussion on possible future research directions, such as context awareness, signal compression, and improved input representations for deep learning algorithms.

## 1. Introduction

Advances in data acquisition, sensing technologies, displays, and extended battery life have allowed for the burgeoning of wearable devices, especially those used for fitness and activity tracking, as well as physiological signal monitoring. Recent projections suggest that by 2026, the wearables market will rise to over 260 billion USD, with over 75 billion USD coming from wristwear (smart watches, smart bracelets) [1]. Wearable devices allow for continuous, unobtrusive, and long-term recording of physiological signals in real-world settings, thus show potential not only for individuals interested in learning more about their health and fitness levels, but also opens doors for new research directions and for, potentially, world-wide experiments.

Recent examples of this new era of patient monitoring could be witnessed during the COVID-19 pandemic, where numerous studies were run on tens of thousands of participants remotely using wearables. Fitbit, for example, showed that prediction of whether a person was sick on any specific day was possible by looking at specific patterns in respiration rate, heart rate, and heart rate variability for that day and the four preceding days [2]. The UCSF study, in turn, showed that the Oura ring could be used to measure heart rate and changes in dermal temperature to predict the onset of COVID-19 symptoms such as fever, cough, and fatigue [3]. The Stanford study, in turn, is currently using wearables to track combinations of step count, body movement, galvanic skin response, temperature, location, steps, calories burned, active minutes, heart rate, the amount of oxygen in the blood, blood pressure, and the quality of sleep in order to predict COVID-19 infections prior to symptoms showing. Pilot analyses have already shown promising results [4].

In addition to COVID-19 infection monitoring, wearables have shown to disrupt numerous healthcare domains, including cardiology [5], gait analysis [6], sleep quality [7], clinical trials during cancer treatment [8], stress management [9], and emotion/depression [10], just to name a few. In addition to healthcare, wearables have also seen applications in smart vehicles [11], pedestrian tracking [12], gaming [13] and extended reality [14], smart homes and robots [15], construction safety [16], and Industry 4.0 applications [17]. There is no doubt that wearables are here to stay.

Wearable devices have gained popularity due to the fact that they allow for measurement in real-world settings in an unobtrusive manner. Previously, measurement of physiological signals was traditionally performed in controlled laboratory conditions with non-ambulant subjects. Although this assures high signal quality and reduces confounding factors, the accuracy of the obtained models can deteriorate when used in highly ecological settings (e.g., see [18]). It is known that ambulant users can generate a large amount of artifacts that can severely affect signal quality [19].

For example, comparisons by [20] between wrist-worn wearable heart rate data and data measured from a chest-worn device showed that correlations were reduced in real-world settings. The work of [21], in turn, showed that movement had an impact on the quality of electrocardiography (ECG) and galvanic skin response (GSR) signals. The work by [22] showed the susceptibility of photoplethysmography (PPG) signals to motion noise, and ultimately, the impact it has on the calculation of heart rate variability measures during daily life movements, especially when strenuous movement is involved [23]. Additionally, the work in [24] showed that motion artifacts may coexist with respiratory movement, thus also making respiratory rate estimation from PPG signals challenging. Electroencephalography (EEG) signals are also known to be inherently sensitive to muscle and eye movements and blink artifacts. Moreover, dry electrodes, typically found in wearable EEG headsets, have shown to have increased sensitivity to movement artifacts relative to wet electrode based systems that are typically found in the clinic [25]. A recent study measuring the reliability and validity of consumer wearable devices for the measurement of heart rate, energy expenditure, and steps in free-living settings showed that (i) high-intensity activity caused significant deterioration in heart rate estimation, (ii) in over 40% of the evaluated studies, step count errors were above the acceptable 10% error margin, and (iii) in 29% of the studies, errors in energy expenditure calculations exceeded the 10% threshold [26].

Although the potential is clear that wearable devices can revolutionize numerous sectors, significant work is still needed in order to measure and enhance the quality of the measured signals. Over the years, numerous methods have been proposed for signal quality measurement and enhancement of different physiological signals and wearable device data. Methods can rely on human intervention [27] or be (semi)automated. Representative examples of the latter include methods that rely on data statistics (e.g., [28,29]), spectral/connectivity profiles (e.g., [30,31,32]), blind source separation (e.g., [33,34]), adaptive filtering (e.g., [35,36]), and, more recently, on machine and deep learning approaches (e.g., [37,38,39,40]). Combinations of multiple such approaches have also been proposed (e.g., [41,42]). For wearable data and real-time analytics, it is imperative that quality measurement and enhancement methods be automated (i.e., not rely on human intervention), require low computational complexity, rely on small amounts of training data, as these can be hard and/or expensive to collect, and be safe against attacks [43]. Ideally, methods should be able to detect the signal quality and adjust the enhancement scheme “on-the-fly” for optimal results. Few existing systems, however, follow this approach, thus leaving ample room for innovation.

In this technical note, we provide an overview of the so-called modulation spectral signal representation developed and used by the authors across numerous applications over the span of the last decade. By measuring the rate-of-change of signal spectral components, the measure is capable of accurately separating signal from noise, thus allowing for accurate measurement and enhancement of numerous different wearable device signals. In particular, the authors have explored the use of this representation for (i) sensor quality assessment, (ii) automated artifact removal, (iii) noise-robust feature extraction, and (iv) new biomarkers development for disease characterization. The goal of this technical note is to provide the reader with an in-depth overview of these developed methods, showcase new applications developed and how they compared to state-of-the-art methods, as well as open-source toolboxes developed in-house, and finally, provide guidance on potential future work that can leverage the proposed representation. Although the majority of the work presented here has already been published elsewhere, this report will provide readers with a “one-stop-shop” document covering a decade’s worth of research by the authors on the use of the modulation spectrum signal representation for “in the wild” applications involving wearables. For a more in-depth comparison between the developed tools and state-of-the-art methods, the interested reader is referred to the numerous references cited herein.

The remainder of this technical note is organized as follows. First, Section 2 will present the modulation spectral signal representation and its signal-noise separation properties, thus motivating quality measurement and enhancement applications, among others. Section 3 then covers the various wearables applications of the modulation spectrum explored by the authors over the last decade, including a description of in-house developed open-source software. Finally, Section 4 provides a discussion on future research possibilities, and Section 5 concludes the paper.

## 2. Modulation Spectrum Signal Representation

Here, we describe the steps for calculating the modulation spectrum signal representation. Next, we motivate its use for different applications involving wearable devices.

### 2.1. Signal Processing

**Figure 1:**
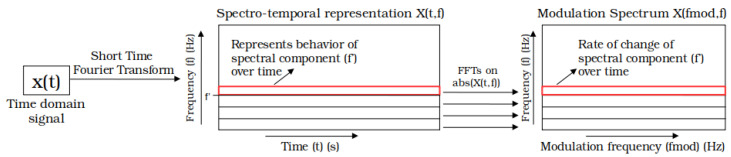
Signal processing steps involved in the computation of a modulation spectrogram. Other methods of computing the representation can be found in [44].

The modulation spectral signal representation measures the rate-of-change of spectral magnitude components. The motivation lies in the fact that, although many artifacts and environmental noise sources overlap in both time and frequency, the rate at which their spectra vary over time differs, hence becoming more separable in the new frequency–frequency modulation spectral domain. As expected, two transformations are needed to obtain the representation; Figure 1 depicts the signal processing steps involved.

First, the time-domain signal x(t) is transformed to its spectrotemporal equivalent X(t,f). This transformation can be achieved by different methods, such as a short-time Fourier transform (STFT), a continuous wavelet transform (CWT), or the use of filterbanks together with the Hilbert transform (HT). These different methods offer certain advantages and disadvantages in terms of time and frequency resolution trade-off. However, for all these methods, the spectrotemporal representation X(t,f) can be written as the convolution of the time-domain signal x(t) and a series of complex filterbanks $\lambda_{f}\left(t\right)e^{j2\pi ft}$, i.e.,

(1) $$X\left(t,f\right)=x\left(t\right)★\lambda_{f}\left(t\right)e^{j2\pi ft},$$

where convolution is indicated with ★, $e^{j2\pi ft}$ denotes complex oscillations for different frequencies *f*, and $\lambda_{f}\left(t\right)$ is the window applied for each complex oscillation at a given frequency. The interested reader is referred to [44] for a detailed description the STFT, CWT, and HT methods, their comparison with each other, and their common formulation in the form of Equation (1).

The next step for the calculation of the modulation spectrogram consists in characterizing in the frequency domain the changes of the instantaneous amplitude for each frequency in the spectrotemporal representation X(t,f). As such, the modulation spectrogram associated to the signal *x* can be written as:

(2) $$X\left(f_{mod},f\right)=F_{t}\left\{|X(t,f)|\right\},$$

where $F_{t}\left\{\cdot \right\}$ indicates the the Fourier transform over the time dimension. This procedure is illustrated in Figure 1, where $f_{mod}$ represents the modulation frequency, whereas *f* represents the conventional frequency axis. Although the phase information of X(t,f) is not used to obtain the modulation spectrogram, phase information is kept if reconstruction of the time-domain signal is needed.

The modulation spectrogram $X(f_{mod},f)$ provides insight into the entire signal’s spectrotemporal dynamics. Such a representation has been found to be useful if stationary noise is present or if desired characteristics from the signal do not change significantly over the short time periods of the recorded signal (e.g., biomarkers of disease over several minute recordings). However, the modulation spectrogram is not time resolved. On the other hand, if transient or time-varying noise is present or time-varying changes in the signal of interest are present (e.g., changing heart rate during exercise), then the modulation spectrogram should be considered in shorter time windows (frames) and window overlaps, hence resulting in a modulation spectral tensor representation of dimension $F\times F_{m}\times N$, where *F* denotes the resolution across the (conventional) frequency axis, $F_{m}$ the resolution across the modulation frequency axis, and *N* the total number of frames available within the signal x(t). It is important to emphasize that the choice of window size and window shifts in the first transform will ultimately dictate the maximum modulation frequency present in the signal, whereas the window size and shifts of the second transform will dictate the resolution of the modulation spectrogram. Different signals require different resolutions and depending on the properties being sought, different frequency ranges are important. As such, these signal processing parameters (e.g., window size, window overlap) are often treated as hyper-parameters and optimized on a per application basis.

Because the modulation spectrum can capture higher order periodicities of the signal, it helps not only to separate signal from noise, but also to quantify properties of the signal not otherwise obvious in time and time–frequency domains. As the authors have shown over the years, the separability of the signal and noise components that is achieved inherently by the method can open doors for numerous innovations. In the sections to follow, we describe the potential of the representation in building new tools for signal quality assessment, signal enhancement, blind source separation, noise-robust feature extraction, and disease characterization. Section 3, in turn, will detail the applications developed by the authors for various different signal modalities recorded from wearable devices, including electrocardiograms (ECG), electroencephalogram (EEG), accelerometry, speech and breath sound recordings, and compare them against existing methods.

### 2.2. Quality Assessment

**Figure 2:**
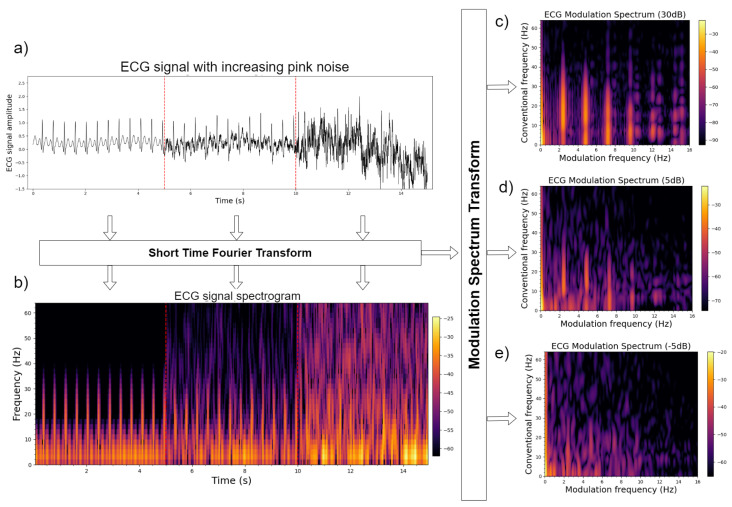
Plots of (**a**) three snippets of synthetic ECG corrupted by noise at SNR levels of 30 dB, 5 dB, and −5 dB; (**b**) their respective time–frequency plots; and (**c**–**e**) the modulation spectrograms for the three signals.

The modulation spectrogram or modulation spectral tensor representation can be used directly for signal quality assessment. As the representation separates signal from noise components, one may characterize the signal and noise components separately and take their ratio to obtain a correlate of the signal-to-noise ratio (SNR) without the need for a clean reference signal. Figure 2 shows an example using a synthetic ECG signal. The top left plot depicts the time-domain signal for the same ECG signal corrupted by recorded sensor noise at three separate SNR levels (30 dB, 5 dB, and −5 dB). The bottom left plot, in turn, depicts the time–frequency representation of the same three signals. Finally, the plots on the right depict the modulation spectrogram of the signals with decreasing SNR from top to bottom. From the top modulation spectrogram plot, it can be seen that ECG information is encoded in the lobes, whereas noise in the areas outside the lobes. By measuring the energy inside and outside the lobes, one can obtain a measure of the signal quality. Quality metrics for ECG and speech signals have been proposed and will be described in more detail in Section 3.

### 2.3. Signal Enhancement

**Figure 3:**
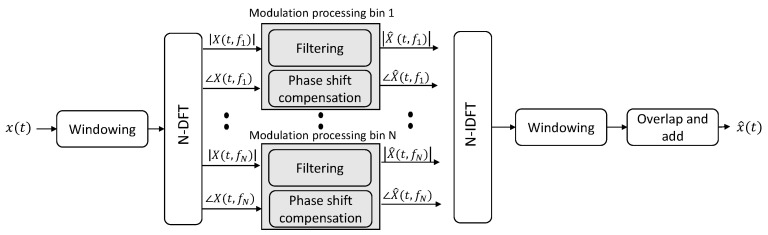
Signal processing steps for signal enhancement via modulation spectrum filtering.

As the modulation spectrum is obtained by performing two time-to-frequency transformations, if such transforms are invertible, one can perform filtering in the modulation spectral domain and invert the filtered representation back to the time domain in order to perform signal enhancement. Figure 3 depicts one such filtering method that performs signal enhancement by keeping the signal components captured by the representation and filtering out the noise components. As will be shown in Section 3.2, different applications will require different filtering schemes. Representative examples include bandpass filterbanks for ECG, bandpass filter for speech, and bandstop filters for EEG. As the filtering can result in phase delays, these need to be accounted for during reconstruction, as shown in the figure.

### 2.4. Blind Source Separation

With physiological data, physiological “noises” are commonly observed and often considered to be nuisance factors, hence are filtered out. Examples of such physiological noise can include the breathing effects on ECG modulations, heart beats appearing in EEGs, or heart and lung sounds concurrently present in breath sound recordings. Often these nuisance signals overlap in both time and frequency, and conventional tools have been developed to suppress/cancel them, hence resulting in enhancement methods, as noted in the preceding section. Indeed, blind signal/source separation differs from signal enhancement in the sense that the interfering signal (e.g., noise) is not filtered out completely, but instead is separated from the signal of interest and used in downstream applications. Separating them in a manner that allows for the nuisance factor to be useful is a far more challenging task. The modulation spectrum representation, in turn, lends itself well to the task as the rate-of-change of the overlapping signals differ, hence become separable in the modulation spectral domain. Such blind source separation of confounding physiological signals can be very useful for wearables, as it allows for additional signal modalities to be measured, hence not only improving accuracy, but also making the systems more robust against missing data (e.g., one could recover the heart rate from the EEG noise and use this information when ECG data from a chest monitor are missing).

### 2.5. Noise-Robust Feature Extraction

Up to now, methods to measure and enhance the quality of wearable data have been noted. Ultimately, however, features need to be extracted from the signals and input into machine learning algorithms for automated detection/classification/recognition tasks. One recurring issue with wearable data collected in realistic settings is that the added noise degrades performance, and enhancement can often remove important details from the signal. One way to bypass the need for enhancement is to derive noise-robust features and use these directly for classification. The modulation spectral representation is an excellent candidate for this, as features can be extracted directly from the signal components in the modulation domain and input to the machine learning pipeline. Noise-robust feature extraction is an important topic for “in-the-wild” studies with wearables, and applications involving ECGs, EEGs, and speech will be highlighted in Section 3.4.

### 2.6. Disease Characterization

Finally, as noted previously, the modulation spectrum representation is useful in quantifying the higher order periodicities of the investigated signals. It turns out that, e.g., disease and aging can affect such periodicities, thus making the modulation spectral signal representation useful for disease characterization. An added bonus is that such differences are often disjoint from the noise spectral dynamics, hence noise-robust diagnostic systems may be possible. Section 3.5 will highlight a few applications explored by the authors, including Alzheimer’s disease diagnosis and severity level prediction, stress and anxiety monitoring, and dysarthric speech intelligibility characterization.

## 3. Applications

Over the last decade, the authors and their collaborators have explored the use of the modulation spectral representation across numerous domains. Here, we highlight those related to wearable devices. The interested reader will be directed to published papers providing more in-depth technical details and comparative analyses with state-of-the-art methods.

### 3.1. Quality Assessment

Due to its ability to separate signal from noise, the modulation spectrum domain has been used to estimate the quality of various signals, as detailed in the subsections to follow.

#### 3.1.1. Electrocardiograms

A modulation spectrum quality index (MS-QI) for ECG quality assessment was proposed in [45]. MS-QI utilizes the insights shown in Figure 2 and proposes the ratio of the total modulation energy inside the lobes to the total modulation energy outside the lobes as a correlate of the ECG SNR. The metric was tested on four different datasets: one of synthetic ECG data contaminated by different noise types at different levels (ranging from 30 dB to −10 dB SNR), and three based on recorded ECGs under varying physical activities and quality levels. Experimental results showed the proposed metric outperforming conventional ECG quality benchmarks, including ECG kurtosis and the in-to-out-of-band spectral power ratio within the QRS complexes, across all tested conditions. More recently, in [46], a variant of the MS-QI metric was proposed based on signals measured from an instrumented steering wheel and showed to achieve results in line with those obtained from a chest ECG monitor. The predicted quality can also be used as context in real-world applications. For example, in [47], the MS-QI metric was used to flag only usable portions of the ECG to be stored on the wearable device for future offline analysis. Such a strategy was able to save 65% in storage requirements and reduce energy requirements. These are crucial gains for wearable devices.

#### 3.1.2. Speech Signals

**Figure 4:**
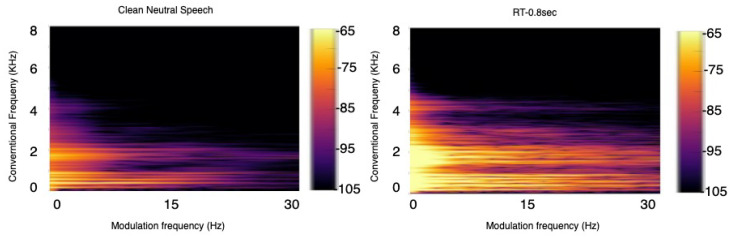
Modulation spectrogram for speech in clean (**left**) and with reverberant ((**right**) RT60 = 0.8 s) environments.

Based on physical constraints imposed by our vocal chords and speech production system, humans are not capable of generating spectral changes beyond certain rates. Experiments conducted over the years have shown that important speech components lie between 2 and 20 Hz modulation frequency [48], and noise and room acoustics can affect such frequencies and/or appear in a different frequency range [49]. Figure 4, for example, depicts the modulation spectrogram of clean speech and speech corrupted by reflections in a medium-sized room with a reverberation time (RT60) of 0.8 s. As can be seen, for clean speech, the majority of the modulation energy lies below 20 Hz $f_{mod}$, while increasing reverberation levels cause increases in energy outside this range.

Using these insights, a blind speech quality and intelligibility measure was proposed in [50] and called speech-to-reverberation modulation energy ratio (SRMR). The measure computes the ratio between the amount of modulation energy below 20 Hz (speech) and the amount of energy above 20 Hz (reverberation). The SRMR metric was shown to not only correlate highly with speech quality but also with room acoustic parameters [51,52]. Extensive experiments showed the proposed metric significantly outperforming several state-of-the-art metrics in use by the International Telecommunications Union (ITU-T), including the so-called P.563, P.862, and P.863 standards. Later work showed that (i) normalizing the metric could improve quality measurement accuracy [53], (ii) incorporating hearing impairments into the SRMR metric could allow for quality assessment for impaired listeners [54], (iii) using the modulation spectral tensor representation could provide more flexibility [55] and serve as input to deep learning algorithms [56], and (iv) incorporating bone-conduction properties could allow for in-ear microphone speech quality monitoring [57]. The SRMR metric was used as a benchmark by many systems in the 2014 REVERB (REverberant Voice Enhancement and Recognition Benchmark) Challenge [58] and the 2015 ACE (Acoustic Characterisation of Environments) Challenge [59]. The work in [60] compared the SRMR metric and its hearing-impairment adjustments to several state-of-the-art methods (including the aforementioned ITU-T standards) across numerous datasets. Experimental results showed the superiority of the method, particularly for cochlear implant users and for hearing aid users with nonlinear frequency compression methods enabled.

### 3.2. Signal Enhancement

Once the regions of the modulation spectrogram tied to signal and those linked to noise have been found, one can perform filtering in the modulation domain for signal enhancement. In the subsections to follow, different enhancement algorithms developed by the authors will be described.

#### 3.2.1. Electrocardiograms

**Figure 5:**
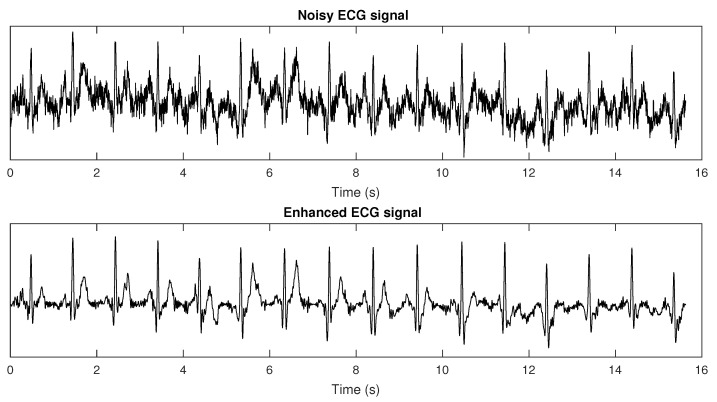
Plots of time domain (**top**) noisy ECG signal (60 bpm) corrupted with an SNR = −5 dB and (**bottom**) its enhanced counterpart obtained after modulation spectrum domain based filtering.

In [61], we proposed the use of a bank of adaptive bandpass filters in the modulation domain in order to keep only the modulation spectrogram lobes responsible for signal information (as per insights from Figure 2). Finite impulse response filters were used, such that phase shifts could be applied during reconstruction to minimize artifacts. Experiments with synthetic and recorded ECGs were performed, and improved heart rate measurement and heart rate variability (HRV) monitoring could be achieved after the proposed enhancement strategy. Results were compared with a state-of-the-art wavelet enhancement algorithm. On the synthetic data, the proposed method was able to reduce heart rate estimation errors from 57% at an SNR of −10 dB to 2.2%, thus outperforming the benchmark, which, in turn, resulted in a 6.2% error rate. Improved HRV measurement was also shown, where errors in the pNN50 metric at extremely noisy cases were around 20% for the benchmark and only 5% for the proposed method. More importantly, the results showed that the improvements in heart rate and heart rate variability measurements could be achieved with negligible computational overhead. Figure 5 depicts a representative example of a noisy ECG corrupted at −5 dB and its corresponding enhanced counterpart obtained via modulation spectrum filtering. More recently, this ECG enhancement strategy was used to enhance very noisy wearable ECG signals and to improve the accuracy of peak detection algorithms, such as the widely-used Pan–Tomkins algorithm [62], under ambulant conditions. The enhanced ECGs were able to better measure heart rate variability and their effects on mental workload [63].

#### 3.2.2. Speech Signals

As noted previously, speech is mostly constrained between 2 and 20 Hz $f_{mod}$, and researchers first proposed the use of low/bandpass filters in the modulation spectrum domain for speech enhancement (e.g., [64,65]). The idea was later applied for environment-robust feature extraction via the so-called RASTA (relative spectral) feature processing pipeline [66]. Although such filtering schemes were shown to indeed remove unwanted noise, it was also observed that unwanted artifacts were introduced. Removal of DC modulation energy can cause issues when applying the inverse transform, as negative amplitude values may result. As the modulation representation is measured from the absolute values, a negative value is not possible. Typically, half-wave rectification was used to counter this issue, hence resulting in unwanted artifacts, such as musical noise. As such, in [67] we proposed the use of bandpass modulation filtering followed by bandwidth expansion in the modulation spectrum domain. In essence, the speech-dominated 2–20 Hz modulation frequency band was used to estimate the speech contribution in the 0–2 Hz band. Filtering combined with bandwidth expansion was shown not only to reduce the musical noise effects to negligible levels, but also to result in enhanced speech that was preferred by the majority of the listeners. For example, when compared against the state-of-the-art noise suppression algorithm at the time (i.e., the one available in the enhanced variable rate codec, EVRC), a subjective listening test showed that 86.25% of the listeners preferred the proposed method over the benchmark. More recently, the concept of multi-microphone beamforming for speech enhancement was proposed in the modulation spectral domain [68]. Improvements of over 7 dB in signal-to-noise ratio improvement relative to conventional beamforming schemes were reported.

#### 3.2.3. Electroencephalograms

Portable EEG signals are contaminated by eye blinks and muscle movement artifacts, among other factors. Similar to the other signals already covered in this technical note, such artifacts vary at a rate that differs from normative EEG. As such, the work in [69] characterized the modulation spectral “patches” related to such artifacts and applied bandstop filters to remove them from EEG signatures of hands, feet, and tongue imagined movements. The enhanced EEGs were then reconstructed and applied to a standard motor imagery brain–computer interface pipeline. An experiment with the BCI Competition IV dataset showed the proposed method outperforming the Challenge winner on six of the nine participants.

### 3.3. Blind Source Separation

As noted previously, blind signal/source separation differs slightly from signal enhancement in the sense that the interfering signal (e.g., noise) is not filtered out completely, but instead is separated from the signal of interest and used in downstream applications. Two applications explored by the authors are detailed next.

#### 3.3.1. Measuring Breathing Rate from ECGs

**Figure 6:**
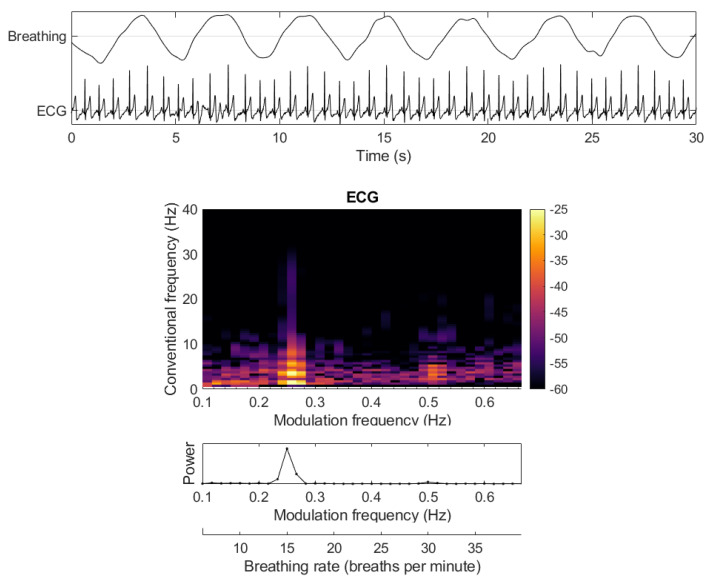
Measuring breathing rate from the breathing-related modulations in an ECG signal.

It is known that breathing modulates the amplitude of the ECG and the changes occur at rates much different that the rates expected from ECGs. Figure 6 depicts the effects that breathing has on the ECG, how this shows up in the modulation spectrogram, and how breathing rate can be recovered. The work in [70] showed that breathing rates could be recovered with high accuracy even from noisy ECG signals, with respiration rate estimation errors below 10%, and with correlations with true breathing rates as high as 0.9. More recently, these results were replicated with sensors embedded in a car steering wheel, and multimodal driver mental state classification could be achieved [46].

#### 3.3.2. Heart and Lung Sound Separation from Breath Sound Recordings

The work in [71] showed that heart and lung sounds measured from breath sound recordings overlapped highly in both time and frequency domains. Hence, state-of-the-art wavelet based separation algorithms still suffered from high levels of residual noise. To overcome this issue, power-complementary bandpass and bandstop filters were applied in the modulation spectral domain to separate heart and lung sounds from the breath sound recordings. After separation, the residual noise was shown to be imperceptible, and both signals were accurately separated even when the breath sound recordings were taken from different locations on the participant’s chest. Comparisons were made with a state-of-the-art wavelet separation method, and the log spectral distance (LSD) was used as a measure of how well the heart and lung sounds were separated. The proposed method achieved an LSD below 0.8 dB, whereas the benchmark resulted in LSD values greater than 1.1 dB. In the audio coding community, LSD values below unity are indicative of processing that results in imperceptible distortions. Moreover, the processing time of the proposed method was shown to be significantly lower for the proposed method, relative to the benchmark (i.e., 2.44 s vs. 67 s by the benchmark to process a 65-second recording).

### 3.4. Noise-Robust Feature Extraction

If machine-based classification is of interest, it may be more efficient (in terms of computational power and energy requirements) to bypass enhancement and extract noise-robust features directly from the modulation spectrogram and apply them to downstream machine learning algorithms. In the subsections below, various different noise-robust features are described.

#### 3.4.1. Electrocardiograms

The work in [72] showed that the modulation spectral signal representation could be used to more accurately measure the “instantaneous” heart rate of very noisy wearable ECG data, thus could be used to more accurately predict heart rate variability (HRV). A new modulation domain HRV metric was proposed and shown to outperform dozens of conventional time, frequency, and non-linear domain HRV metrics across a number of different noisy conditions with and without the use of a wavelet enhancement algorithm. For example, on synthetic noisy data, the correlation between the proposed HRV and the true HRV was of 0.93 at an SNR = −8 dB, whereas for the noisy signal, this correlation was 0.5. Although wavelet enhancement improved results to 0.62, this was still far below the results achieved with the new proposed metric. With this new feature, heart rate errors remained at 1.7% at SNR = −10 dB, thus performing better than when filtering was applied, with both the modulation filter (2.2%) or the wavelet method (6.2%), thus showing that noise-robust feature extraction can be a viable alternative to pre-processing.

#### 3.4.2. Speech Signals

Extraction of noise-robust features from speech modulation spectrograms has been explored for a number of applications by the authors. The work in [73], for example, showed that new features extracted from the 2–20 Hz $f_{mod}$ range could be used for reliable automatic speaker identification in far-field conditions. When compared to a state-of-the-art benchmark based on mel-frequency cepstral coefficients, it was shown that a slight decrease in accuracy was seen in clean conditions relative to the benchmark (i.e., 96.6% vs. 97.2% accuracy), but the accuracy remained unchanged with increasing levels of reverberation. The accuracy of the benchmark system, in turn, quickly dropped to unacceptable values (accuracy below 45%) at higher reverberation levels around RT60 of one second. Similarly, new features were proposed for speech emotion recognition in noise [74], stress detection [75], as well as for whispered speech detection in noise [76], and for whispered speech-based speaker verification [77]. In all applications, comparisons with state-of-the-art benchmarks and features showed the superiority of the proposed methods.

#### 3.4.3. Electroencephalograms

In [78,79], new modulation spectral features were extracted from EEG signals and shown to better correlate with the mental workload and stress ratings provided by ambulant users. The proposed features not only outperformed widely used power spectral subband features, but also showed complementary behavior that boosted overall accuracy when fused together. The work in [80], in turn, showed that new coupling measures computed directly from the EEG modulation spectral representation could better predict user emotional states relative to conventional benchmark spectral, asymmetry, and phase–amplitude coupling measures. Using balanced accuracy as a figure of merit, it was shown that the proposed features could outperform the benchmarks by as much as 8%, 20%, and 6.5% for valence, dominance, and liking ratings, respectively. More recently, the work in [81] showed that amplitude modulation information from the EEG signal, in particular related to alpha band modulations, was correlated with cortical hemodynamics measured via functional near-infrared spectroscopy (fNIRS), as well as that it could predict the performance of an fNIRS-based affective brain-computer interface [82].

#### 3.4.4. Accelerometry

**Figure 7:**
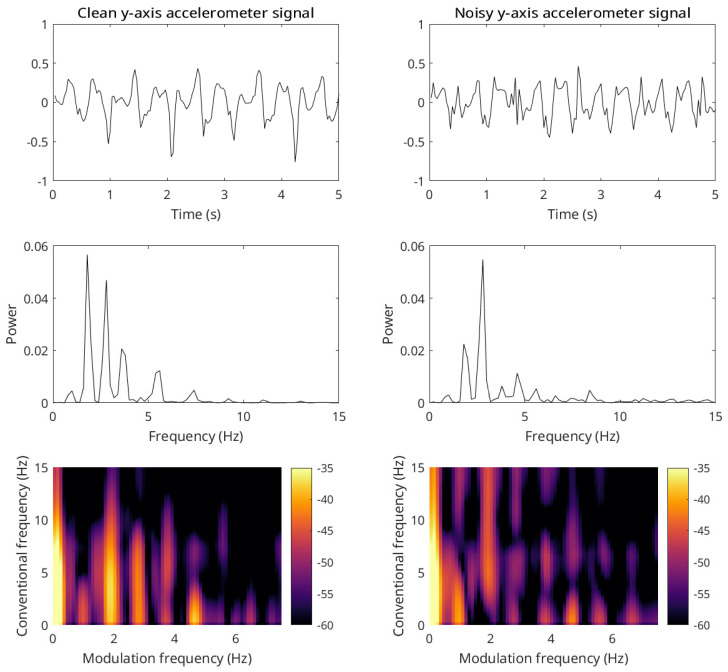
Time domain representation (**top plots**) of a clean (**left**) and noisy (**right**) *y*-axis accelerometer signal segment (sampled at 30 Hz) and their corresponding spectra (**middle plots**) and modulation spectrograms (**bottom plots**).

The work in [83] showed that wearable accelerometers could be used to measure the gait speed of elderly patients performing a 400 m walk test. The recorded signals were shown to be too noisy for conventional gait speed calculations based on widely used kinematic models. Figure 7 shows two representative signals, with the one on the left representing a cleaner accelerometry signal and the one on the right a noisier one. As can be seen, conventional spectrum-based measures are not capable of capturing the stride information (spectral peak) accurately in the noisier case, whereas the modulation spectral representation is able to preserve those details even under very noisy conditions. Experimental results showed the proposed gait speed measure outperforming benchmark measures across a number of different testing conditions. For example, a correlation of 0.98 was obtained between the proposed measure and the true gait speed measures; for comparison, the kinematic model benchmark achieved a correlation of 0.68. Further tests on the MAREA (Movement Analysis in Real-world Environments using Accelerometers) dataset [84] confirmed a correlation of 0.99 with ground truth speeds.

### 3.5. Disease Characterization

Disease is also known to affect hidden periodicities in different signal modalities. This neuromodulatory deficit was first shown in EEG signals with patients with Alzheimer’s disease (AD) [85]. Wearable systems with automated enhancement algorithms were later explored [86] and further optimized for portability and low-cost [87]. Features were extracted from the EEG modulation spectrogram based on frequency subbands widely used in the EEG literature and compared to conventional measures extracted from these subbands, including magnitude/phase coherence and spectral power based benchmarks. Results showed the proposed features outperforming these benchmarks by 8%, 5%, and 11% in terms of accuracy, sensitivity, and specificity, respectively. Gains further improved when the proposed measures were fused with the benchmarks, resulting in improvements of 9% in accuracy and sensitivity. More recently, in [88], we showed that alternate subbands were more useful, in terms of both conventional and modulation frequency dimensions, and resulted in more robust accuracy. For example, the new measures achieved an F1-score of 0.75 using raw EEG data, thus outperforming the previous modulation spectrum measured based on conventional bands (F1 = 0.70) and a benchmark based on spectral features (F1 = 0.69). Although accuracy gains could be seen after enhancement was applied, the gains were not significant (F1 = 0.76), thus suggesting that the new features are also more robust to EEG artifacts. These new so-called modulation spectral “patches” were obtained via visual inspection. More recently, they have also been validated and improved upon with machine learning principles based on the use of saliency maps obtained with convolutional neural networks [89]. The new data-driven patches have resulted in further gains and have shown the importance of higher gamma band frequencies.

**Figure 8:**
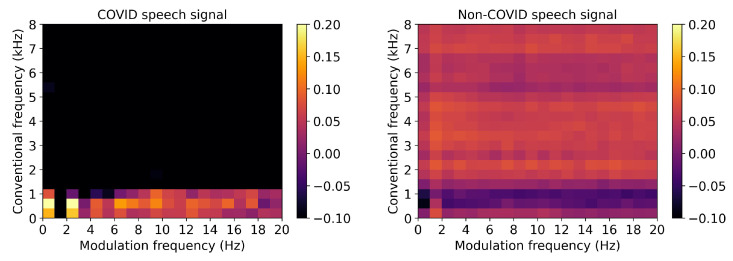
Normalized average modulation spectrograms for speech made by individuals with COVID-19 (**left**) and by healthy individuals (**right**).

From speech, dysarthria is known to affect speech intelligibility and the amplitude modulation of the produced speech sounds. The work in [90] proposed a new intelligibility measure for dysarthric speech and showed that the new measure outperformed several measures widely used in the clinic, including prosody and nasality ones. Actual severity levels were later predicted by the measures in [91]. In [92], modulation spectral features were also shown to discriminate toddlers diagnosed with autism spectrum disorder based on the analysis of the amplitude modulation of their cries and non-verbal vocalizations. Experimental results showed the proposed features outperforming several prosodic features and achieving results in line with a state-of-the-art wavelet based benchmark. Fusion experiments showed further improvements, thus suggesting their complementarity. More recently, the modulation spectrum has shown to be a useful tool for COVID-19 infection detection based on speech. As an illustration, Figure 8 depicts the normalized average modulation spectrogram for speech made by individuals diagnosed with COVID-19 (left) and by healthy individuals (right). Preliminary analyses suggest that features extracted from the modulation spectrum can accurately detect COVID-19 infection. Experiments with the INTERSPEECH 2021 COVID-19 detection challenge dataset show the proposed method significantly outperforming the Challenge benchmark system [93].

### 3.6. In-House Developed Software

**Figure 9:**
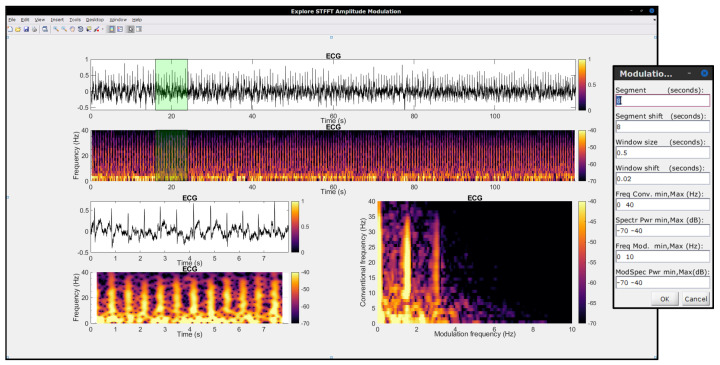
A screenshot of the in-house developed open-source. Amplitude Modulation Analysis (AMA) Toolbox user interface. The toolbox can be used for modulation spectral signal analysis.

In order to help advance science and to facilitate the replication of research results, it is desirable for open-source code to be available to the community. The University of Washington Modulation Matlab Toolbox [94] has been available since the early 2000s and has been widely used by the authors, especially in earlier work involving speech and audio signals. An in-house toolbox for the SRMR speech quality metric was developed and made available at our Lab’s GitHub page (https://github.com/MuSAELab, accessed on 15 May 2022) under the *SRMRToolbox* repository. The normalized and hearing-impaired user adaptations noted in Section 3.1.2 are also available. More recently, a faster quantized version of the modulation spectrogram has been implemented and made available under the *modulation_filterbanks* repository. These toolboxes, however, have been tailored to acoustic signals, hence a gap existed when it came to analyzing biological signals. As such, the Amplitude Modulation Analysis (AMA) toolbox was developed and made available to the community in Matlab/Octave (under repository *amplitude-modulation-analysis-matlab*) and Python (*amplitude-modulation-analysis-module*) programming languages. More details about the toolbox can be found in [95]; Figure 9 shows a screenshot of the AMA toolbox user interface. For completeness and replication purposes, the interested reader can use the *modulation-spectrogram-technical-note* repository and associated readme files and scripts to regenerate all of the images included in this technical note.

## 4. Future Research Possibilities

Artificial intelligence and machine learning tools are burgeoning, and wearables-based applications have started to emerge for healthcare, human performance monitoring, smart cities, and automated systems, to name a few. Many of the used algorithms, however, have been developed with computer vision applications in mind, hence rely on 2-dimensional image-like inputs (e.g., convolutional deep neural networks). To this end, the spectrogram has played a crucial role in enabling the use of these algorithms with 1-dimensional time series data, such as biosignals and speech, hence generating the image-like inputs required by the deep neural networks. As shown here, the modulation spectrogram can serve as a powerful alternative to the conventional spectrogram, as it can better separate signal and noise components. Such improved discrimination can help reduce the complexity of the classification algorithms, as shown by [96] for a mental state classification task based on heart rate measured from a wearable. This can have a direct effect on minimizing the environmental impact of the machine learning algorithms as well as mitigating privacy and security concerns by allowing models to be run directly on portable devices (edge computing). As such, this technological report has only described a very small subset of possibilities that can be achieved with the modulation spectrogram.

The possibilities for future research directions are numerous and may include the development of new tools and applications across different verticals, including the exploration of the use of the modulation spectrogram with other signal modalities (e.g., electrooculography, electromyography). Moreover, the modulation spectrum can be used to measure context from the separated noise components and provide this information to machine learning algorithms. Currently, accelerometers have been widely used to characterize movements, and tools based on wireless sensing are emerging (e.g., [97,98]). With the modulation spectrum, however, we have shown in [72] that sitting, walking, and running could be discriminated directly from the noise patterns measured from the ECG modulation spectrogram. Such contextual information can provide additional intelligence to machine learning algorithms.

An additional potential avenue is in the detection of so-called adversarial attacks, where carefully crafted noise can be added to input signals to force the classifier to make mistakes while yet being very confident in their decisions. Adversarial attacks can have drastic outcomes, especially in healthcare applications. Typically, these noises are made to be imperceptible to the user, but the modulation spectrum may help sift out such attacks. Moreover, by separating signal from noise, potentially improved compression algorithms can be developed by focusing only on the signal components. In fact, such an approach may lead to combined compression-and-enhancement schemes for 1-dimensional signals, such as speech and biosignals. Such approaches have been proposed for images (e.g., [99]) but lack for other time series based signals. Finally, future work could explore the use of the modulation spectrogram as an alternative input modality to emerging deep learning algorithms. This could lead to new state-of-the-art results as well as the generation of new biomarkers of disease (e.g., as in [89] for Alzheimer’s disease diagnosis). It is hoped that by making open-source tools easily accessible to the research community, barriers to achieving these innovations can be reduced.

## 5. Conclusions

This technical note has presented the modulation spectral signal representation that has been proposed and used by the authors over the last decade across numerous “in the wild” applications involving wearable devices. Applications in signal quality measurement and enhancement, blind source separation, robust feature extraction, and disease characterization have been highlighted. Future research possibilities have also been highlighted, and existing open-source tools are listed to facilitate research replication and the development of new applications. The goal of this technical note was to compile all of the research outcomes obtained by the authors using this particular technology, thus serving as a “one stop shop” for researchers interested in building reliable applications using wearables in highly ecological settings.

## Data Availability

Not applicable.
